# *trans*-4-(1-Naphth­yl)-2-oxo-1,3-oxazolidine-5-carboxylic acid

**DOI:** 10.1107/S1600536808019132

**Published:** 2008-07-12

**Authors:** Jiao-Yan Yang, Zhi-Hui Ming, Jing An, Qiu-Lin Hua, Liang-Qiu Lu

**Affiliations:** a College of Life Sciences, Central China Normal University, Wuhan 430079, People’s Republic of China; b Key Laboratory of Pesticides and Chemical Biology of the Ministry of Education, College of Chemistry, Central China Normal University, Wuhan 430079, People’s Republic of China

## Abstract

The crystal structure of the title compound, C_14_H_11_NO_4_, is influenced by N—H⋯O and O—H⋯O hydrogen bonds, linking mol­ecules into one-dimensional tapes running along the [010] direction.

## Related literature

For general backgroud regarding the title compound, see: Lu *et al.* (2008 ▶). For patterns in hydrogen bonding, see: Bernstein *et al.* (1995 ▶). For related literature, see: Barbachyn & Ford (2003 ▶); Evans (1982 ▶); Mukhtar & Wright (2005 ▶).
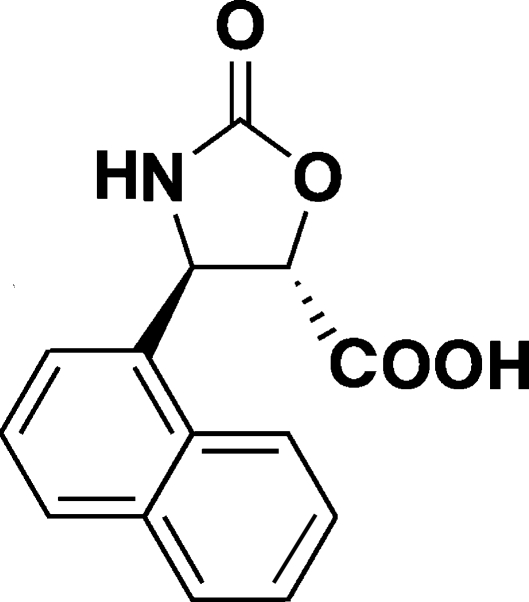


## Experimental

### 

#### Crystal data

C_14_H_11_NO_4_
*M* *_r_* = 257.24
Orthorhombic, 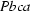

*a* = 8.7159 (17) Å
*b* = 12.817 (3) Å
*c* = 20.737 (4) Å
*V* = 2316.6 (8) Å^3^
*Z* = 8
Mo *K*α radiation
μ = 0.11 mm^−1^
*T* = 292 (2) K
0.47 × 0.38 × 0.35 mm

#### Data collection

Bruker SMART 4K CCD area-detector diffractometer
Absorption correction: none
12484 measured reflections
2266 independent reflections
2008 reflections with *I* > 2σ(*I*)
*R*_int_ = 0.016

#### Refinement

*R*[*F* ^2^ > 2σ(*F* ^2^)] = 0.036
*wR*(*F* ^2^) = 0.093
*S* = 1.04
2266 reflections
178 parameters
H atoms treated by a mixture of independent and constrained refinement
Δρ_max_ = 0.19 e Å^−3^
Δρ_min_ = −0.20 e Å^−3^

### 

Data collection: *SMART* (Bruker, 2001 ▶); cell refinement: *SAINT* (Bruker, 2001 ▶); data reduction: *SAINT*; program(s) used to solve structure: *SHELXS97* (Sheldrick, 2008 ▶); program(s) used to refine structure: *SHELXL97* (Sheldrick, 2008 ▶); molecular graphics: *PLATON* (Spek, 2003 ▶); software used to prepare material for publication: *PLATON*.

## Supplementary Material

Crystal structure: contains datablocks I, global. DOI: 10.1107/S1600536808019132/bg2191sup1.cif

Structure factors: contains datablocks I. DOI: 10.1107/S1600536808019132/bg2191Isup2.hkl

Additional supplementary materials:  crystallographic information; 3D view; checkCIF report

## Figures and Tables

**Table 1:** Hydrogen-bond geometry (Å, °)

*D*—H⋯*A*	*D*—H	H⋯*A*	*D*⋯*A*	*D*—H⋯*A*
O1—H1*A*⋯O4^i^	0.94 (2)	1.72 (2)	2.6591 (15)	174 (2)
N1—H1⋯O2^ii^	0.834 (17)	2.247 (17)	3.0097 (18)	152.0 (15)

Symmetry codes: (i) 
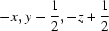
; (ii) 
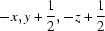
.

**Fig. 1.**
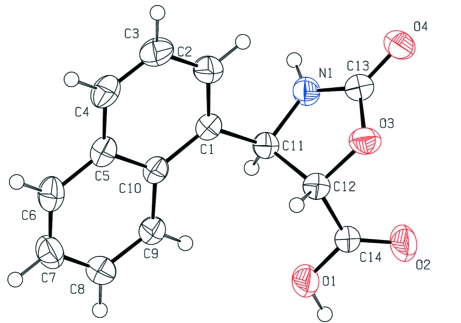
View of the molecule of (I) showing the atom-labelling scheme. Displacement ellipsoids are drawn at the 50% probability level.

**Fig. 2.**
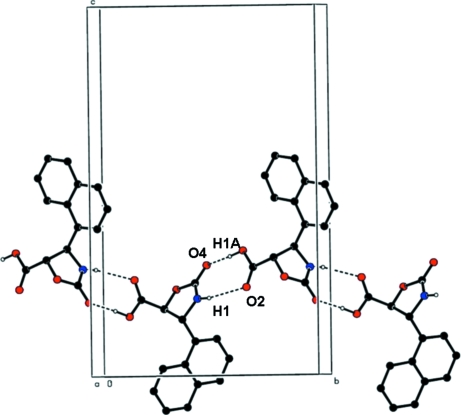
Packing of (I) with hydrogen bonds drawn as dashed lines showing the formation of hydrogen-bonded R22(8) loops involving NH···O and OH···O hydrogen bonds. H atoms not involved in hydrogen bonds have been omitted for clarity. [Symmetry codes: (i) -x, y - 1/2, -z + 1/2; (ii) -x, y + 1/2, z + 1/2]

## supplementary crystallographic information

### Comment

Oxazolidin-2-ones are prevalent in biologically active molecules (Mukhtar *et al.*, 2005; Barbachyn, *et al.*, 2003), as well as versatile synthons in organic synthesis (Evans, 1982). Recently we reported a new cascade reaction to synthesize these compounds from stable sulfur ylides and nitroolefins (Lu *et al.*, 2008). In order to demostrate the utility of this method we continued to hydrolyze *trans*-ethyl 4-(naphthalen-1-yl)-2-oxooxazolidine-5-carboxylate with LiOH to obtain *trans*-4-(naphthalen-1-yl)-2-oxooxazolidine-5-carboxylic acid, and we are presenting herein the X-ray crystallographic analysis of the title compound, thus obtained (Fig. 1),

The crystal structure is determined by N—H···O and O—H···O hydrogen bonds, defining R_2_^2^(8) rings (Bernstein *et al.* (1995), which link molecules into one-dimensional hydrogen-bonded tapes along [010] ( Fig. 2).

### Experimental

The starting material, 4-(naphthalen-1-yl)-2-oxooxazolidine-5-carboxylate (100.0 mg, 0.35 mmol) was added to the aqueous solution of LiOH (88.1 mg, 2.10 mmol, 2.5 ml H2O) and the reaction mixture was stirred for 2.5 h. By adjusting the pH = 6–7 with concentrated HCl and 1*M* diluted HCl solution, the white solid precipitated and was filtrated. The residue was washed with cold water and diethyl ester, the desired product was collected as white power after dryness with 93% yield. Recrystallization from CH3OH—H2O provided the crystalline solid.

### Refinement

All H atoms bonded to C atoms were initially located in difference Fourier maps and then constrained to their ideal geometry positions with C–H=0.96Å (methyl), 0.97Å (methylene). H atoms bonded to N and O were found in difference maps and refined with N/O—H distances free. In all cases *U*_iso_ ((H) values were set to x times *U*_eq_(host), x= 1.5(methyl), x=1.2 (methylene), x=1.2 (N), x=1.5 (O).

### Crystal data

**Table:**

C_14_H_11_NO_4_	*F*_000_ = 1072
*M**_r_* = 257.24	*D*_x_ = 1.475 Mg m^−^^3^
Orthorhombic, *P**b**c**a*	Mo *K*α radiation λ = 0.71073 Å
Hall symbol: -P 2ac 2ab	Cell parameters from 5223 reflections
*a* = 8.7159 (17) Å	θ = 2.8–28.9º
*b* = 12.817 (3) Å	µ = 0.11 mm^−^^1^
*c* = 20.737 (4) Å	*T* = 292 (2) K
*V* = 2316.6 (8) Å^3^	Block, colorless
*Z* = 8	0.47 × 0.38 × 0.35 mm

### Data collection

**Table:**

Bruker SMART 4K CCD area-detector diffractometer	2008 reflections with *I* > 2σ(*I*)
Radiation source: fine-focus sealed tube	*R*_int_ = 0.016
Monochromator: graphite	θ_max_ = 26.0º
*T* = 292(2) K	θ_min_ = 3.0º
φ and ω scans	*h* = −9→10
Absorption correction: none	*k* = −15→15
12484 measured reflections	*l* = −25→25
2266 independent reflections	

### Refinement

**Table:**

Refinement on *F*^2^	Secondary atom site location: difference Fourier map
Least-squares matrix: full	Hydrogen site location: inferred from neighbouring sites
*R*[*F*^2^ > 2σ(*F*^2^)] = 0.036	H atoms treated by a mixture of independent and constrained refinement
*wR*(*F*^2^) = 0.093	*w* = 1/[σ^2^(*F*_o_^2^) + (0.0416*P*)^2^ + 0.7823*P*] where *P* = (*F*_o_^2^ + 2*F*_c_^2^)/3
*S* = 1.05	(Δ/σ)_max_ < 0.001
2266 reflections	Δρ_max_ = 0.19 e Å^−^^3^
178 parameters	Δρ_min_ = −0.20 e Å^−^^3^
Primary atom site location: structure-invariant direct methods	Extinction correction: none

### Special details

**Table:**

Geometry. All e.s.d.'s (except the e.s.d. in the dihedral angle between two l.s. planes) are estimated using the full covariance matrix. The cell e.s.d.'s are taken into account individually in the estimation of e.s.d.'s in distances, angles and torsion angles; correlations between e.s.d.'s in cell parameters are only used when they are defined by crystal symmetry. An approximate (isotropic) treatment of cell e.s.d.'s is used for estimating e.s.d.'s involving l.s. planes.
Refinement. Refinement of *F*^2^ against ALL reflections. The weighted *R*-factor *wR* and goodness of fit *S* are based on *F*^2^, conventional *R*-factors *R* are based on *F*, with *F* set to zero for negative *F*^2^. The threshold expression of *F*^2^ > σ(*F*^2^) is used only for calculating *R*-factors(gt) *etc*. and is not relevant to the choice of reflections for refinement. *R*-factors based on *F*^2^ are statistically about twice as large as those based on *F*, and *R*- factors based on ALL data will be even larger.

### Fractional atomic coordinates and isotropic or equivalent isotropic displacement parameters (Å^2^)

**Table:**

	*x*	*y*	*z*	*U*_iso_*/*U*_eq_	
C1	0.10060 (15)	0.89669 (10)	0.40612 (6)	0.0322 (3)	
C2	0.18399 (17)	0.98662 (11)	0.40013 (7)	0.0415 (3)	
H2	0.1971	1.0159	0.3595	0.050*	
C3	0.25014 (19)	1.03567 (11)	0.45381 (7)	0.0474 (4)	
H3	0.3063	1.0967	0.4484	0.057*	
C4	0.23264 (17)	0.99464 (12)	0.51357 (7)	0.0438 (4)	
H4	0.2764	1.0280	0.5489	0.053*	
C5	0.14847 (15)	0.90147 (11)	0.52271 (6)	0.0363 (3)	
C6	0.13034 (17)	0.85684 (12)	0.58449 (7)	0.0435 (4)	
H6	0.1731	0.8900	0.6201	0.052*	
C7	0.05170 (18)	0.76650 (13)	0.59291 (7)	0.0468 (4)	
H7	0.0424	0.7377	0.6339	0.056*	
C8	−0.01545 (17)	0.71659 (12)	0.53978 (7)	0.0435 (3)	
H8	−0.0694	0.6548	0.5458	0.052*	
C9	−0.00239 (15)	0.75784 (10)	0.47936 (6)	0.0359 (3)	
H9	−0.0490	0.7242	0.4448	0.043*	
C10	0.08091 (14)	0.85104 (10)	0.46843 (6)	0.0315 (3)	
C11	0.03291 (15)	0.84401 (10)	0.34692 (6)	0.0326 (3)	
H11	−0.0684	0.8149	0.3567	0.039*	
C12	0.13968 (15)	0.75872 (10)	0.31821 (6)	0.0356 (3)	
H12	0.2150	0.7374	0.3507	0.043*	
C13	0.13627 (16)	0.89576 (10)	0.24897 (7)	0.0370 (3)	
C14	0.05709 (17)	0.66298 (10)	0.29259 (6)	0.0390 (3)	
N1	0.02505 (13)	0.91215 (9)	0.29122 (5)	0.0363 (3)	
H1	−0.0281 (18)	0.9662 (13)	0.2897 (7)	0.044*	
O1	−0.01994 (14)	0.61525 (9)	0.33777 (5)	0.0538 (3)	
H1A	−0.068 (3)	0.5549 (17)	0.3222 (10)	0.081*	
O2	0.0653 (2)	0.63355 (10)	0.23870 (5)	0.0869 (5)	
O3	0.21709 (11)	0.80945 (7)	0.26568 (5)	0.0429 (3)	
O4	0.17042 (13)	0.94603 (8)	0.20100 (5)	0.0497 (3)	

### Atomic displacement parameters (Å^2^)

**Table:**

	*U*^11^	*U*^22^	*U*^33^	*U*^12^	*U*^13^	*U*^23^
C1	0.0341 (6)	0.0298 (6)	0.0328 (7)	0.0010 (5)	0.0002 (5)	−0.0034 (5)
C2	0.0503 (8)	0.0350 (7)	0.0394 (7)	−0.0056 (6)	0.0002 (6)	0.0012 (6)
C3	0.0529 (9)	0.0346 (8)	0.0545 (9)	−0.0125 (6)	−0.0010 (7)	−0.0056 (6)
C4	0.0452 (8)	0.0422 (8)	0.0439 (8)	−0.0054 (6)	−0.0044 (6)	−0.0138 (6)
C5	0.0358 (7)	0.0378 (7)	0.0353 (7)	0.0037 (6)	−0.0010 (5)	−0.0071 (6)
C6	0.0452 (8)	0.0538 (9)	0.0316 (7)	0.0033 (7)	−0.0036 (6)	−0.0071 (6)
C7	0.0544 (9)	0.0542 (9)	0.0319 (7)	0.0063 (7)	0.0019 (6)	0.0065 (6)
C8	0.0505 (8)	0.0383 (8)	0.0415 (8)	−0.0020 (6)	0.0059 (6)	0.0035 (6)
C9	0.0402 (7)	0.0339 (7)	0.0337 (7)	−0.0017 (6)	0.0008 (5)	−0.0038 (5)
C10	0.0322 (6)	0.0306 (6)	0.0317 (6)	0.0032 (5)	0.0004 (5)	−0.0042 (5)
C11	0.0363 (7)	0.0314 (7)	0.0302 (6)	−0.0008 (5)	−0.0008 (5)	−0.0002 (5)
C12	0.0421 (7)	0.0314 (7)	0.0333 (7)	0.0010 (6)	0.0005 (6)	0.0009 (5)
C13	0.0407 (7)	0.0313 (7)	0.0389 (7)	0.0004 (6)	0.0004 (6)	0.0004 (6)
C14	0.0563 (9)	0.0307 (7)	0.0301 (7)	−0.0004 (6)	0.0037 (6)	−0.0003 (5)
N1	0.0398 (6)	0.0369 (6)	0.0322 (6)	0.0083 (5)	0.0001 (5)	0.0023 (5)
O1	0.0767 (8)	0.0478 (6)	0.0370 (6)	−0.0233 (6)	0.0127 (5)	−0.0085 (5)
O2	0.1626 (15)	0.0612 (8)	0.0371 (6)	−0.0509 (9)	0.0266 (8)	−0.0154 (6)
O3	0.0437 (5)	0.0328 (5)	0.0521 (6)	0.0043 (4)	0.0128 (5)	0.0052 (4)
O4	0.0620 (7)	0.0415 (6)	0.0457 (6)	0.0059 (5)	0.0143 (5)	0.0106 (5)

### Geometric parameters (Å, °)

**Table:**

C1—C2	1.3682 (19)	C8—H8	0.9300
C1—C10	1.4289 (17)	C9—C10	1.4160 (18)
C1—C11	1.5202 (17)	C9—H9	0.9300
C2—C3	1.403 (2)	C11—N1	1.4497 (16)
C2—H2	0.9300	C11—C12	1.5542 (18)
C3—C4	1.355 (2)	C11—H11	0.9800
C3—H3	0.9300	C12—O3	1.4368 (16)
C4—C5	1.414 (2)	C12—C14	1.5185 (19)
C4—H4	0.9300	C12—H12	0.9800
C5—C6	1.4117 (19)	C13—O4	1.2220 (16)
C5—C10	1.4253 (18)	C13—N1	1.3233 (18)
C6—C7	1.357 (2)	C13—O3	1.3565 (16)
C6—H6	0.9300	C14—O2	1.1817 (17)
C7—C8	1.402 (2)	C14—O1	1.3049 (17)
C7—H7	0.9300	N1—H1	0.834 (17)
C8—C9	1.3647 (19)	O1—H1A	0.94 (2)
			
C2—C1—C10	119.40 (12)	C9—C10—C5	117.90 (12)
C2—C1—C11	120.46 (12)	C9—C10—C1	123.49 (11)
C10—C1—C11	120.12 (11)	C5—C10—C1	118.61 (12)
C1—C2—C3	121.59 (13)	N1—C11—C1	113.21 (11)
C1—C2—H2	119.2	N1—C11—C12	98.44 (10)
C3—C2—H2	119.2	C1—C11—C12	112.92 (11)
C4—C3—C2	120.38 (13)	N1—C11—H11	110.6
C4—C3—H3	119.8	C1—C11—H11	110.6
C2—C3—H3	119.8	C12—C11—H11	110.6
C3—C4—C5	120.58 (13)	O3—C12—C14	108.85 (10)
C3—C4—H4	119.7	O3—C12—C11	104.67 (10)
C5—C4—H4	119.7	C14—C12—C11	114.74 (11)
C6—C5—C4	121.45 (13)	O3—C12—H12	109.5
C6—C5—C10	119.12 (13)	C14—C12—H12	109.5
C4—C5—C10	119.43 (12)	C11—C12—H12	109.5
C7—C6—C5	121.27 (13)	O4—C13—N1	129.31 (13)
C7—C6—H6	119.4	O4—C13—O3	120.76 (12)
C5—C6—H6	119.4	N1—C13—O3	109.92 (12)
C6—C7—C8	119.93 (13)	O2—C14—O1	124.10 (14)
C6—C7—H7	120.0	O2—C14—C12	124.05 (13)
C8—C7—H7	120.0	O1—C14—C12	111.81 (11)
C9—C8—C7	120.66 (14)	C13—N1—C11	113.40 (11)
C9—C8—H8	119.7	C13—N1—H1	120.9 (11)
C7—C8—H8	119.7	C11—N1—H1	123.9 (11)
C8—C9—C10	121.10 (12)	C14—O1—H1A	111.7 (13)
C8—C9—H9	119.5	C13—O3—C12	108.59 (10)
C10—C9—H9	119.5		
			
C10—C1—C2—C3	−0.4 (2)	C2—C1—C11—N1	−17.73 (18)
C11—C1—C2—C3	−178.76 (13)	C10—C1—C11—N1	163.91 (11)
C1—C2—C3—C4	0.0 (2)	C2—C1—C11—C12	93.05 (15)
C2—C3—C4—C5	0.4 (2)	C10—C1—C11—C12	−85.31 (14)
C3—C4—C5—C6	179.30 (15)	N1—C11—C12—O3	21.29 (12)
C3—C4—C5—C10	−0.4 (2)	C1—C11—C12—O3	−98.41 (12)
C4—C5—C6—C7	−178.87 (14)	N1—C11—C12—C14	−97.95 (12)
C10—C5—C6—C7	0.8 (2)	C1—C11—C12—C14	142.36 (11)
C5—C6—C7—C8	−1.0 (2)	O3—C12—C14—O2	4.2 (2)
C6—C7—C8—C9	0.1 (2)	C11—C12—C14—O2	121.12 (18)
C7—C8—C9—C10	0.9 (2)	O3—C12—C14—O1	−177.87 (12)
C8—C9—C10—C5	−1.09 (19)	C11—C12—C14—O1	−61.00 (16)
C8—C9—C10—C1	178.86 (13)	O4—C13—N1—C11	−171.98 (14)
C6—C5—C10—C9	0.22 (19)	O3—C13—N1—C11	7.85 (16)
C4—C5—C10—C9	179.92 (12)	C1—C11—N1—C13	101.23 (13)
C6—C5—C10—C1	−179.73 (12)	C12—C11—N1—C13	−18.24 (14)
C4—C5—C10—C1	−0.03 (19)	O4—C13—O3—C12	−172.25 (13)
C2—C1—C10—C9	−179.53 (13)	N1—C13—O3—C12	7.90 (15)
C11—C1—C10—C9	−1.16 (19)	C14—C12—O3—C13	104.23 (12)
C2—C1—C10—C5	0.42 (19)	C11—C12—O3—C13	−18.91 (13)
C11—C1—C10—C5	178.79 (11)		

### Hydrogen-bond geometry (Å, °)

**Table:**

*D*—H···*A*	*D*—H	H···*A*	*D*···*A*	*D*—H···*A*
O1—H1A···O4^i^	0.94 (2)	1.72 (2)	2.6591 (15)	174 (2)
N1—H1···O2^ii^	0.834 (17)	2.247 (17)	3.0097 (18)	152.0 (15)

Symmetry codes: (i) −*x*, *y*−1/2, −*z*+1/2; (ii) −*x*, *y*+1/2, −*z*+1/2.
